# Fucoxanthinol, Metabolite of Fucoxanthin, Improves Obesity-Induced Inflammation in Adipocyte Cells

**DOI:** 10.3390/md13084799

**Published:** 2015-08-04

**Authors:** Hayato Maeda, Shogo Kanno, Mei Kodate, Masashi Hosokawa, Kazuo Miyashita

**Affiliations:** 1 Faculty of Agriculture and Life Science, Hirosaki University, 3 Bunkyo-cho, Hirosaki, Aomori 036-8561, Japan; E-Mails: kannoshogo1020@yahoo.co.jp (S.K.); kmsnooo0710@yahoo.co.jp (M.K.); 2 Faculty of Fisheries Sciences, Hokkaido University, 3-1-1 Minato, Hakodate, Hokkaido 041-8611, Japan; E-Mails: hoso@fish.hokudai.ac.jp (M.H.); kmiya@fish.hokudai.ac.jp (K.M.)

**Keywords:** fucoxanthin, fucoxanthinol, adipocyte, obesity, inflammation

## Abstract

Fucoxanthin (Fx) is a marine carotenoid found in edible brown seaweeds. We previously reported that dietary Fx metabolite into fucoxanthinol (FxOH), attenuates the weight gain of white adipose tissue of diabetic/obese KK-*A^y^* mice. In this study, to evaluate anti-diabetic effects of Fx, we investigated improving the effect of insulin resistance on the diabetic model of KK-*A^y^* mice. Furthermore, preventing the effect of FxOH on low-grade chronic inflammation related to oxidative stress was evaluated on 3T3-L1 adipocyte cells and a RAW264.7 macrophage cell co-culture system. A diet containing 0.1% Fx was fed to diabetic model KK-*A^y^* mice for three weeks, then glucose tolerance was observed. Fx diet significantly improved glucose tolerance compared with the control diet group. In *in vitro* studies, FxOH showed suppressed tumor necrosis factor-α (TNF-α), and monocyte chemotactic protein-1 (MCP-1) mRNA expression and protein levels in a co-culture of adipocyte and macrophage cells. These findings suggest that Fx ameliorates glucose tolerance in the diabetic model mice. Furthermore, FxOH, a metabolite of Fx, suppresses low-grade chronic inflammation in adipocyte cells.

## 1. Introduction

Obesity is regarded as a major risk factor for type-2 diabetes, hypertension, and dyslipidemia [1]. The cluster is called metabolic syndrome, of which the incidence is a worldwide problem [2]. Obesity is characterized by low-grade chronic inflammation; it is the mechanism responsible for insulin resistance caused by obesity [3].

Adipocytokines, such as TNF-α and MCP-1, are biologically-active mediators secreted from adipocytes cells. They are related to the development of insulin resistance and are associated with low-grade inflammation throughout the body [4,5]. Especially, MCP-1 induces a vicious cycle that increases inflammatory responses in obesity by causing monocyte/macrophage accumulation and activation in adipose tissues [6]. Therefore, agents that can suppress adipose tissue macrophage behavior might prevent obesity-related diseases. Furthermore, oxidative stress contributes to the pathogenesis of insulin resistance and type-2 diabetes [7,8]. Reactive oxygen species secreted from adipocytes cells promote insulin resistance in various organs. Thus, antioxidants protect oxidative stress in adipocytes cells, ameliorating insulin resistance [9].

Fucoxanthin (Fx) is a characteristic carotenoid of brown seaweeds, including edible species such as *Laminaria japonica* and *Undaria pinnatifida*. They are among the most popular food ingredients of Japanese cuisine. Fx shows anti-cancer, anti-inflammatory, and radical scavenging activity [10,11,12,13]. Furthermore, it shows anti-obesity and anti-diabetic effects [14,15,16,17,18,19]. Especially, dietary Fx-induced expression of uncoupling protein 1 (UCP1) plays an important role on energy expenditure in white adipose tissue (WAT) [14,15]. UCP1 protein is expressed exclusively in brown adipose tissues (BAT). However, most of the human adult adipose tissue is WAT; BAT is present in the body only in minute amounts. UCP1 expression in WAT is an attractive target for the development of anti-obesity therapies [20,21]. Due to this unique activity, Fx has attracted much attention for use as a new functional food and ingredient.

Dietary Fx also regulates adipocytokine secretion and prevents hyperglycemia in type-2 diabetes model mice [15,22]. However, its active component remains unknown. Dietary Fx is hydrolyzed to FxOH in the gastrointestinal system [23]. The metabolites accumulate in the internal organs, such as liver and adipose tissue [24]. It is, therefore, important to ascertain whether Fx metabolites, FxOH, act directly on WAT composed of adipocytes and/or macrophages. An earlier study demonstrated that FxOH prevents effects of 3T3-L1 pre-adipocyte differentiation. Its effect is stronger than that of Fx [25]. Additionally, FxOH suppressed pro-inflammatory mediator expression in RAW264.7 macrophage cells and 3T3-L1F442A adipocyte cells [22]. However, the studies only determined each cell, not the real response *in vivo*. In addition, dietary Fx ameliorated blood glucose levels resulting in sufficient reduction of WAT weight in obese mouse [26]. Preventing the effects of low-grade chronic inflammation of dietary Fx is important, rather than suppressing the accumulation of WAT.

This study investigated the effect of Fx on glucose tolerance in diabetic model mice. Furthermore, to assess whether FxOH can inhibit low-grade chronic inflammation in an adipocyte inflammation model efficiently, or not, a co-culture of adipocytes and macrophages system was used for experimentation.

## 2. Results

### 2.1. Animal Experiment

Body weight and food intake were not different between the control group and the Fx 0.1% group. The WAT weight tended to be suppressed in the Fx 0.1% group compared with the control group (control group 9.00 ± 0.41 g/100 g body weight, Fx 0.1% group 7.66 ± 0.46 g/100 g body weight). Brown adipose tissue (BAT) weight was increased in the Fx 0.1% group compared with the control group, although not significantly (control group 0.52 ± 0.02 g/100 g body weight, Fx 0.1% group 0.81 ± 0.04 g/100 g body weight). Other tissue weights were not differentiated among the experimental groups.

**Figure 1:**
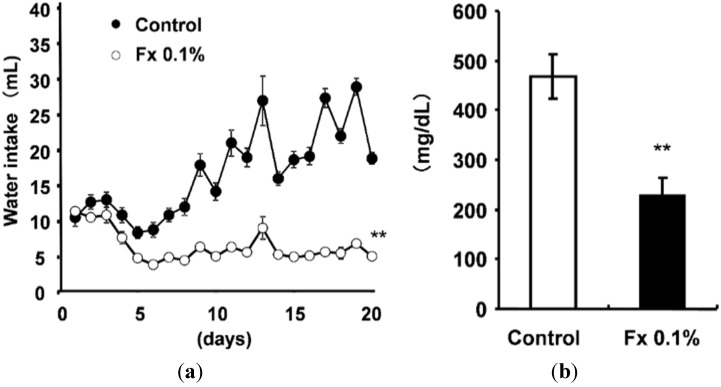
(**a**) Water intake on KK-*A^y^* mice fed experimental diets; (**b**) Plasma blood glucose level of KK-*A^y^* mice. Each value is the mean ± SE (*n* = 6). ** *p* < 0.01 *vs.* Control.

**Figure 2:**
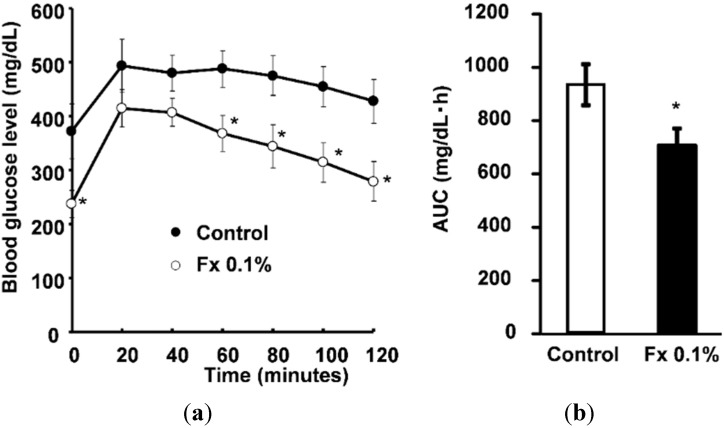
(**a**) Blood glucose level of glucose tolerance test on KK-*A^y^* mice fed with experimental diets; (**b**) AUC level of blood glucose level on the glucose tolerance test. Each value is the mean ± SE (*n* = 6). * *p* < 0.05 *vs.* Control.

Water intake was suppressed significantly in the Fx 0.1% group compared with control group (Figure 1a). Furthermore, the blood glucose level improved significantly in the Fx 0.1% group compared with the control group (Figure 1b). Figure 2 presents results of glucose tolerance tests. After administration of glucose (2 mg/g of body weight), the glucose level of the mice of the Fx 0.1% group was significantly (*p* < 0.05) lower than that of the control group 60, 80, 100, and 120 min after administration (Figure 2a). Figure 2b shows the area under the curve (AUC) level of the blood glucose level. The Fx 0.1% group blood glucose level recovered rapidly compared with that of the Control group.

**Figure 3:**
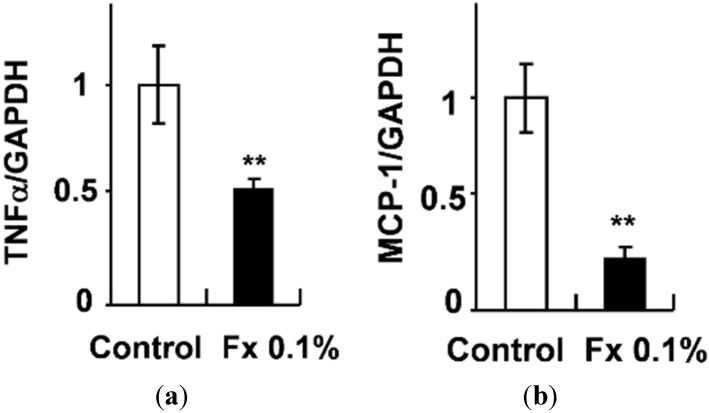
(**a**) Tumor necrosis factor-α (TNF-α) mRNA expressions in WAT of KK-*A^y^* mice; (**b**) Monocyte chemoattractant protein-1 (MCP-1) mRNA expressions in WAT of KK-*A^y^* mice. Each value is the mean ± SE (*n* = 6). ** *p* < 0.01 *vs.* Control.

Levels of mRNA expressions related to low-grade chronic inflammation in WAT were measured using real-time quantitative RT-PCR (Figure 3). The TNF-α and MCP-1 mRNA expressions were significantly suppressed (*p* < 0.01) in the Fx 0.1 groups compared to control groups.

### 2.2. Effects of FxOH on the Induction of Inflammatory Changes by Co-Culture of Adipocytes and Macrophages

**Figure 4:**
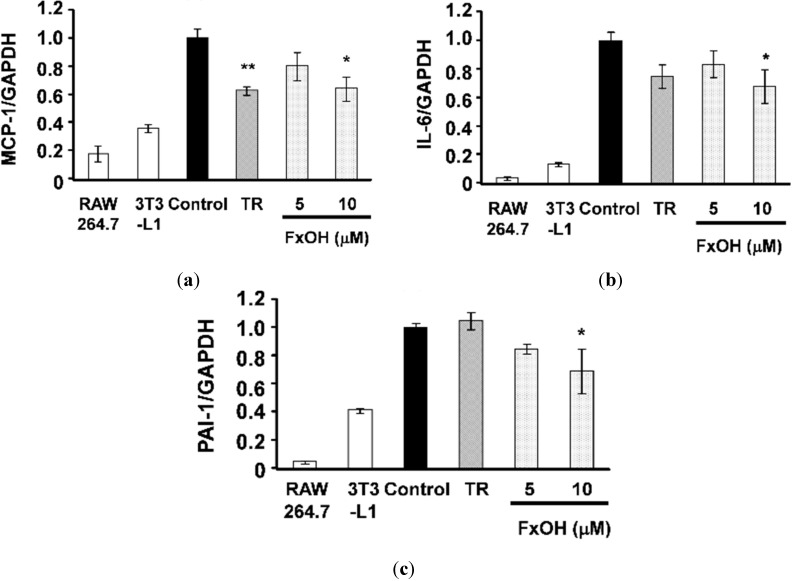
MCP-1, IL-6, PAI-1 mRNA expressions in a co-culture of 3T3-L1 adipocytes and RAW 264.7 macrophage cells. Co-cultured cells were incubated with 10 μM troglitazone (TR) or 5, 10 μM FxOH for 24 h: (**a**) MCP-1; (**b**) IL-6; (**c**) PAI-1. Each value is the mean ± SE (*n* = 4). * *p* < 0.05 *vs.* Control; ** *p* < 0.01 *vs.* control.

**Figure 5:**
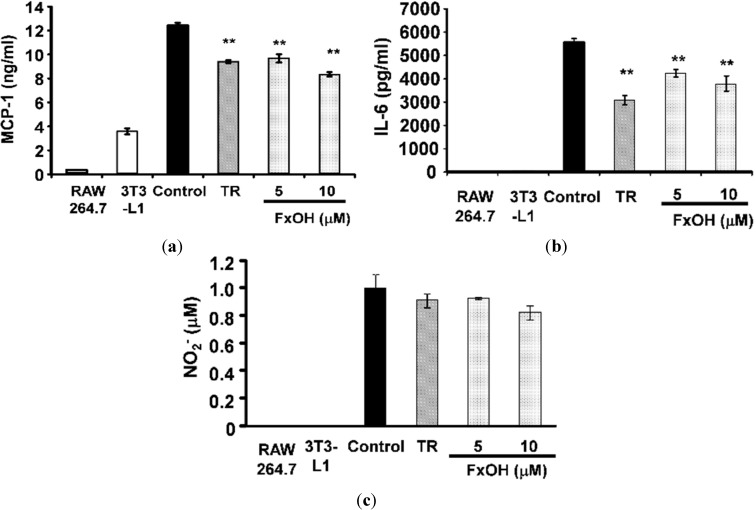
Effects of FxOH on MCP-1, IL-6 protein secretion and NO secretion in a co-culture of 3T3-L1 adipocyte cells and RAW264.7 macrophage cells system. Co-culture cells were incubated with 10 μM troglitazone (TR) or 5, 10 μM FxOH for 24 h: (**a**) MCP-1; (**b**) IL-6; (**c**) NO. * *p* < 0.05 *vs.* control.

The major adipocytokines MCP-1, interleukin-6 (IL-6), and plasminogen activator inhibitor-1 (PAI-1) promote inflammatory action in adipocytes. A co-culture of 3T3-L1 adipocytes and RAW264.7 macrophage cells (Control) increased these adipocytokines’ mRNA expression compared with cultured cells of each alone. Treatment with 10 μM troglitazone (TR), which is a peroxisome proliferator-activated receptor γ (PPARγ) ligand agonist, suppressed MCP-1 mRNA expression in the co-culture system (Figure 4a). Treatment with 10 μM FxOH cells significantly suppressed (*p* < 0.05) MCP-1, IL-6, and PAI-1 mRNA expression compared to that in control cells (Figure 4a–c). Moreover, cells treated with FxOH showed significantly decreased (*p* < 0.01) MCP-1 and IL-6 production, dose-dependently (Figure 5a,b). NO production was not significantly lower, but it tended to be suppressed when cells were treated with FxOH (Figure 5c).

**Figure 6:**
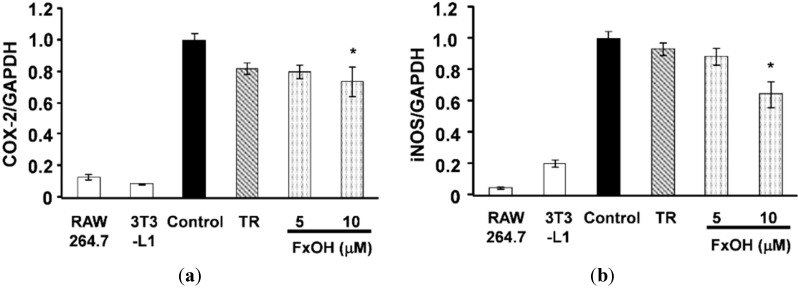
Effects of FxOH on COX-2, iNOS mRNA expression in a co-culture of 3T3-L1 adipocyte cells and RAW264.7 macrophage cells system. Co-culture cells were incubated with 10 μM troglitazone (TR) or 5, 10 μM FxOH for 24 h: (**a**) COX-2; (**b**) iNOS. * *p* < 0.05 *vs.* control.

**Figure 7:**
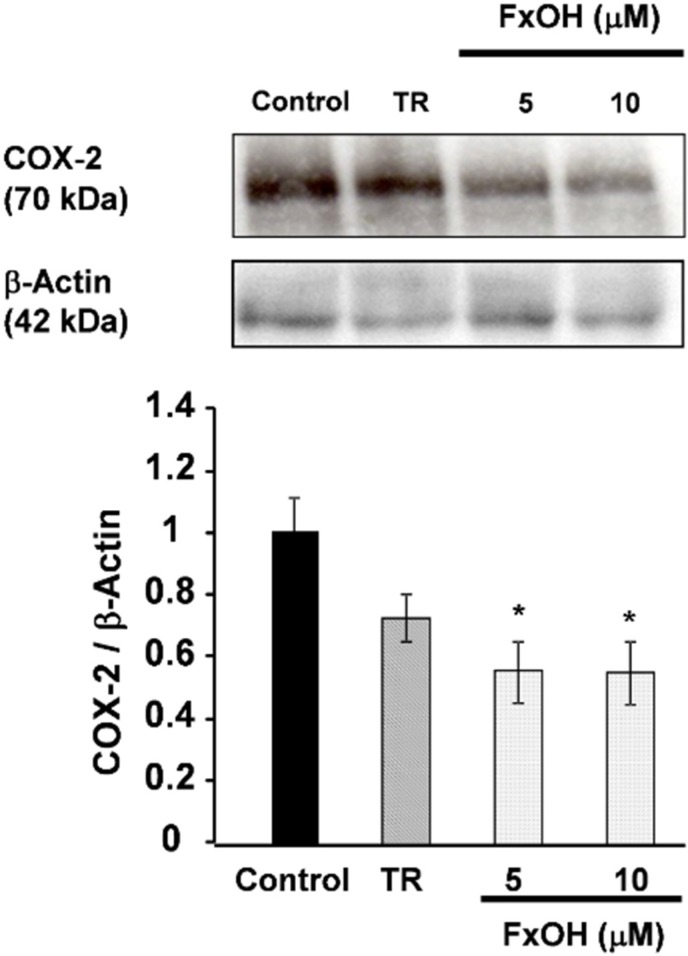
Effects of FxOH on COX-2 protein expression in a co-culture of 3T3-L1 adipocyte cells and RAW264.7 macrophage cells system. Co-culture cells were incubated with 10 μM troglitazone (TR) or 5, 10 μM FxOH for 24 h. * *p* < 0.05 *vs.* control.

The cyclooxygenase-2 (COX-2) and inducible nitric oxide synthase (iNOS) mRNA expressions were markedly down-regulated (*p* < 0.05) after treatment with 10 μM FxOH cells compared to control cells (Figure 6). In addition, COX-2 protein expressions in cells were determined using Western blot analysis. Cells treated with FxOH (5, 10 μM) exhibited suppressed COX-2 protein expression compared with that of control cells (Figure 7). These results suggest that FxOH ameliorates inflammatory changes in adipocyte cells induced by macrophage migration.

## 3. Discussion

This study demonstrated the effect of Fx for anti-diabetic treatment and whether FxOH, a metabolite of Fx, can also act as an anti-inflammatory agent in the interaction of adipocytes and macrophages.

In fact, Fx ameliorates disorders of blood glucose levels in diabetic model KK-*A^y^* mice. Moreover, oral glucose tolerance tests show that Fx improves glucose intolerance. This effect is regarded as down-regulation of pro-inflammatory mediators such as TNF-α and MCP-1 in WAT. Based on those results, the anti-inflammatory effect of dietary Fx on adipocyte cells was examined.

Results show that FxOH suppressed MCP-1, IL-6, and PAI-1 mRNA expressions in a co-culture of adipocytes and macrophages. Furthermore, FxOH suppressed production of MCP-1, IL-6, and NO secretion in co-culture cells. The effect is similar to that of TR, which is a PPARγ activation ligand, such as thiazolidinedione. Thiazolidinedione improves insulin sensitivity by increasing concentrations of adiponectin, and down-regulating inflammatory factor TNF-α levels [27,28]. Recent reports have described obesity as an inflammatory disease that causes insulin resistance in adipose tissues, skeletal muscle, and the liver [29]. Obese adipose tissues are characterized by enhanced infiltration of macrophages. A paracrine loop involving adipocyte is believed to derive free fatty acids [30]. Actually, IL-6, MCP-1, resistin, and TNF-α are associated strongly with obesity-induced inflammation and obesity-related pathologies. Adipocyte cell medium has been reported to enhance the expression of TNF-α in macrophages. This induction is regarded as resulting from free fatty acids derived from adipocytes. Free fatty acids released from adipocytes by the lipolysis of triglyceride exert its pro-inflammatory effect on macrophages through the activation of Toll-like receptor 4 (TLR4). Actually, TLR4 is regarded as a necessary cell surface receptor for the recognition of LPS macrophages. Free fatty acids released from adipocytes by the lipolysis of triglycerides exert pro-inflammatory effects on macrophages through the activation of TLR4 [31]. TLR4 regulates several transcription factors encoding inflammatory mediators. Therefore, FxOH probably regulates TLR4 signaling of macrophages and recovers chronic inflammation in adipocyte cells induced by metabolic disorder. Further studies are necessary to clarify the anti-inflammatory pathway of FxOH.

NO produced from activated macrophages is associated with acute and chronic inflammation. Treatment with FxOH suppressed NO production in co-culture cells. In addition, FxOH suppressed iNOS and COX-2 mRNA expression and COX-2 protein expression. iNOS is the key enzyme producing large amounts of NO by macrophages. Increased NO production has been implicated as a cause of diverse inflammatory diseases including rheumatoid arthritis and ulcerative colitis. COX-2 promotes prostaglandin E2 synthesis from arachidonic acid. COX-2 is an inducible enzyme expressed in inflammation-related cells, such as macrophages, producing large amounts of prostaglandins. Excess production of prostaglandins causes inflammatory disorders. The results presented from this study demonstrate that FxOH treatment attenuates inflammation in obesity-induced inflammatory adipocyte cells. However, the effectiveness of FxOH on adipocytes or macrophage cells is unclear. Anti-inflammatory effects of Fx on macrophage cells had already been reported [11,32]. Therefore, it infers anti-inflammatory effects of FxOH on each cell. To reveal this point, further studies need to indicate FxOH effectiveness on adipocytes or macrophage cells.

Reportedly, β-carotene accumulation in 3T3-L1 adipocytes suppresses gene expression related to insulin sensitivity [33]. Our previous study elucidated the similar effects of capsanthin and capsorubin, important constituents of paprika carotenoids [34]. Especially, capsorubin isolated from paprika is a more effective anti-oxidant than β-carotene [35]. Furthermore, FxOH showed strong anti-oxidation activity compared with other carotenoids [36]. Reportedly, anti-oxidant (*N*-acetylcysteine) prevents obesity-induced metabolic changes in 3T3-L1 adipocytes [9]. Therefore, the anti-oxidative activity of FxOH might be related to the attenuation of inflammation in obese adipocytes.

Algae lipid containing Fx, such as *Undaria pinnatifida* or *U.* *sargasso,* show decreasing effects of blood glucose levels in obese mice [14,15,16,17,18,19]. Our previous study showed that Fx decreases WAT weight and that it is related to UCP1 protein expression in WAT [14,15]. UCP1 is normally expressed only in BAT, not in WAT. BAT is related to energy and heat production in tissues by the contribution of UCP1 [20,21]. Therefore, UCP1 expression in WAT has been regarded as a main reason for the anti-obesity and anti-diabetic effect of Fx. In this study, KK-*A^y^* mice fed with a 0.1% Fx diet exhibited decreased blood glucose levels. However, 0.1% administration Fx did not significantly reduce white adipose weight. Nevertheless, Fx diet group improved the blood glucose level. Therefore, the effect of Fx on the glucose level is not only in decreasing the WAT weight; it is also related to changing of the adipocyte properties. Additionally, it can be presumed that FxOH, the metabolite, ameliorates inflammation in adipose tissue and exhibits anti-diabetic effects.

## 4. Materials and Methods

### 4.1. Chemical Reagents and Cells

Mouse 3T3-L1 preadipocyte cells and RAW 264.7 cells were obtained from DS Pharma Biomedical Co. Ltd. (Osaka, Japan). Fetal bovine serum (FBS) was purchased from Biological Industries Ltd. (Kibbutz, Beit, Israel). Dulbecco’s modified Eagle’s medium (DMEM) was purchased from Nissui Pharmaceutical Co. Ltd. (Tokyo, Japan). TR was purchased from Funakoshi Co. Ltd. (Tokyo, Japan). All other chemicals, guaranteed to be of reagent or tissue-culture grade, were from Sigma-Aldrich (St. Louis, Missouri, USA) or Wako Pure Chemical Industries Ltd. (Osaka, Japan).

### 4.2. Fx and FxOH Preparation

Lipid extracts containing fucoxanthin were obtained from commercial *Undaria pinnatifida* dried seaweed using acetone extraction. Fx was purified from the lipid extracts using silica gel column chromatography with *n*-hexane/acetone (7:3, v/v). The purity of Fx (all-*trans*-fucoxanthin + cisfucoxanthin) was >97%, as shown by HPLC analysis. FxOH was hydroxylated by enzyme reaction, as described in previous reports [25].

### 4.3. Animal Care

All procedures for the use and care of animals for this research were approved by the Ethical Committee of Experimental Animal Care at Hokkaido University. Male KK-*A^y^* mice (3 weeks of age; CLEA Japan, Inc., Tokyo, Japan) were housed at 23 ± 1 °C and at 50% humidity with a 12 h light/12 h dark cycle. Each group of mice, six animals had free access to drinking water and a prepared diet. After acclimation for one week, the mice were provided with the experimental diet. The diet compositions are shown in Table 1, prepared according to recommendations of the American Institute of Nutrition (AIN-93G) [37]. After feeding with the experimental diets for 27 days, mice were starved for 12 h and were anatomized under anesthesia. Abdominal WAT and BAT were rapidly removed and weighed. Samples were also taken for mRNA expression analysis and were stored in RNA later® Solution (Sigma-Aldrich Chemical Co., St. Louis, Missouri, USA).

### 4.4. Oral Glucose Tolerance Test

Glucose tolerance tests were conducted one week before the experimental periods. Mice were starved for 7 h, 1.5 g/kg body weight of glucose ingested. After ingestion, blood glucose levels were determined at 0, 20, 40, 60, 80, 100, and 120 min using a blood glucose monitor (Glutest Neo Sensor; Sanwa Kagaku Kenkyusho Co. Ltd., Nagoya, Japan). AUC level was calculated using the trapezoidal rule.

**Table 1:** Composition of experimental diets used in animal experiment (g per kg of diet).

Ingredients	Control	Fx 0.1%
Soybean oil ^1^	135.10	134.10
Fucoxanthin		1.00
Corn starch ^2^	346.28	346.28
Casein ^2^	216.00	216.00
Dextrinized cornstarch ^2^	114.99	114.99
Sucrose ^3^	87.12	87.12
AIN-93 mineral mixture ^2^	35	35
AIN93 vitamin mixture ^2^	10	10
l-cystine ^4^	3	3
Choline bitartrate ^3^	2.5	2.5
Cellulose ^2^	50	50
*tert*-Butylhydroquinone ^4^	0.014	0.014
Total (g)	1000	1000

Note: ^1^ Wako Pure Chemical Ind., Osaka, Japan; ^2^ CLEA Japan, Inc., Tokyo, Japan; ^3^ Kanto Chemical Co., Inc., Tokyo, Japan; ^4^ SIGMA-ALDRICH, St. Louis, Missouri, USA.

### 4.5. Cell Culture

The 3T3-L1 adipocyte cells and RAW264.7 macrophage cells were co-cultured in a contact system. 3T3-L1 preadipocyte cells and RAW 264.7 macrophage cells were cultured, respectively, in DMEM with 10% FBS, 100 U/mL penicillin, and 100 μg/mL streptomycin at 37 °C, in a humidified atmosphere of 95% air and 5% CO2. Differentiation of 3T3-L1 preadipocytes was conducted as described in an earlier report [25]. After 3T3-L1 cells reached confluence, they were incubated for an additional 24 h. Then adipocyte differentiation of 3T3-L1 preadipocytes was initiated, using differentiation medium I, containing 10 μg/mL insulin, 0.5 mmol/L isobutylmethylxanthine, and 0.1 μmol/L dexamethazone for 48 h. The medium was then replaced with DMEM containing 5 μg/mL insulin (differentiation medium II), and changed to fresh medium every 48 h for fresh medium II. After 3T3-L1 cells were cultured in differentiation medium II for 144 h, then RAW 264.7 cells (5 × 10^4^ cells/well) were plated onto 24-well-plate cultured differentiated 3T3-L1 cells. They were treated with DMEM medium containing FxOH or TR for 24 h. Subsequently, cells and culture medium supernatants were collected. FxOH or TR were added as a dimethyl sulfoxide solution. The final concentration of dimethyl sulfoxide was adjusted to 0.1% to avoid affecting cell growth. The cytotoxicity of FxOH on 3T3-L1 cells was determined using WST-1 assay [38].

### 4.6. mRNA Analysis

Total RNA was extracted from mouse uterine WAT (RNeasy^®^ Lipid tissue Mini Kit; Qiagen Inc., Tokyo, Japan) according to the kit manufacturer’s protocol. Then, cDNA was synthesized from total RNA using a kit (High-Capacity cDNA Archive Kit; Applied Biosystems Japan Ltd., Tokyo, Japan). Real-time quantitative RT-PCR analysis was performed with an automated sequence detection system (ABI Prism 7500; Applied Biosystems Japan, Ltd., Tokyo, Japan). The PCR cycling conditions were 40 cycles of 95 °C for 15 s and 60 °C for 1 min. TNF-α, MCP-1, and GAPDH mRNA expression were measured using Taqman® Gene Expression Assays (Applied Biosystems Japan, Ltd., Tokyo, Japan). The PCR primers were purchased (TNF-α, Mm00445641_m1; MCP-1, Mm99999056_m1; GAPDH, Mm99999915_g1; Applied Biosystems Japan Ltd., Tokyo, Japan). Each PCR reaction was normalized to GAPDH.

Total RNA was extracted from culture cells (Quick Gene mini 80; Fujifilm Corp., Tokyo, Japan) according to the manufacturer’s protocol. Then, cDNA was synthesized using the method described above. MCP-1, IL-6, PAI-1, COX-2, and iNOS mRNA expressions were measured using a real-time PCR detection system (Opticon; Bio-Rad Laboratories Inc., Hercules, CA, USA.) and Thunderbird^®^ qPCR Mix (Toyobo Co. Ltd., Osaka, Japan). The primer sequences used for RT-PCR were the following: 5′-CAT GGC CTT CCG TGT TCC TA-3′ (forward) and 5′-GCG GCA CGT CAG ATC CA-3′ (reverse) for mouse GAPDH; 5′-CTG AAG CCA GCT CTC TCT TCC T-3′ (forward) and 5′-CAG GCC CAG AAG CAT GAC A-3′ (reverse) for mouse MCP-1; 5′-CCA CGG CCT TCC CTA CTT C-3′ (forward) and 5′-TTG GGA GTG GTA TCC TCT GTG A-3′ (reverse) for mouse IL-6; 5′-CCG TGG AAC AAG AAT GAG ATC AG-3′ (forward) and 5′-CTC TAG GTC CCG CTG GAC AA-3′ (reverse) for mouse PAI-1; 5′-CTC TAG GTC CCG CTG GAC AA -3′ (forward) and 5′-CTC TAG GTC CCG CTG GAC AA-3′ (reverse) for mouse COX-2; 5′-CTC TAG GTC CCG CTG GAC AA-3′ (forward) and 5′-CTC TAG GTC CCG CTG GAC AA-3′ (reverse) for mouse iNOS. Each PCR reaction was normalized to GAPDH.

### 4.7. Measurement of MCP-1 and IL-6 Production

Concentrations of MCP-1 and IL-6 in the culture supernatants were determined using ELISA, conducted using a Mouse MCP-1 ELISA Kit (thermo Fisher Scientific K.K., Kanagawa, Japan) and Mouse IL-6 ELISA Kit (Takara Bio Inc., Shiga, Japan) in accordance with the manufacturer’s instructions. The amount of nitrite in cell-free culture supernatants was measured using Griess method [34].

### 4.8. Western Blot Analysis

Co-cultured differentiated 3T3-L1 cells and RAW-264.7 cells treated with each sample were lysed with cold RIPA buffer (pH 7.4) containing 20 mmol/L Tris-HCl, 150 mmol/L NaCl, 1% NP-40, 0.5% sodium deoxycholate, 0.1% sodium dodecyl sulfate (SDS), 0.1 mg/mL phenylmethylsulfonyl fluoride, 50 μg/mL aprotinin, and 1 mmol/L Na_3_VO_4_. Cell lysates were centrifuged at 12,000 rpm for 20 min at 4 °C. Then the supernatant (30 μg protein/lane) was separated by 10% SDS-polyvinylidene difluoride membrane. The membrane was incubated with an antibody against iNOS for 1 h and then with secondary antibody rabbit IgG-conjugated horseradish peroxidase (Santa Cruz Biotechnology Inc., Dallas, Texas USA) for 1 h at room temperature. The membranes were treated with reagents (Chemi-Lumi One L; Nacalai Tesque Inc., Kyoto, Japan) according to the manufacturer’s instructions. β-Actin was used as the control with the anti-β-Actin antibody (Santa Cruz Biotechnology Inc., St. Louis, MO, USA).

### 4.9. Statistical Analysis

The results were expressed as mean ± standard error (S.E.). Statistical analyses between multiple groups were conducted using ANOVA. Statistical comparisons were made using Dunnett’s multiple comparison tests. Differences were inferred as significant for *p* < 0.05. Analyses were conducted using software (Stat View-J ver. 5.0; SAS Institute Inc., Cary, IL, USA).

## 5. Conclusions

Fx, and its metabolite FxOH, attenuate inflammatory changes in the interaction between adipocytes and macrophages. These results suggest that Fx contained in edible algae is useful as a food ingredient for controlling obesity-related insulin resistance and for preventing metabolic syndrome.
